# Nonbacterial thrombotic endocarditis as the sole manifestation of stage IV gastric cancer: a case report

**DOI:** 10.1186/1752-1947-8-267

**Published:** 2014-08-04

**Authors:** Wassim Shatila, Alain Rizkallah, Ehab Saad Aldin, Arafat Tfayli

**Affiliations:** 1 Department of Internal Medicine, Duke University, 1020 Goldmist Lane, Durham, North Carolina 27713, USA; 2 Department of Internal Medicine, American University of Beirut Medical Center, PO Box 11-0236, Riad El-Solh 1107-2020 Beirut, Lebanon; 3 Medstar Good Samaritan Hospital, 5601 Loch Raven Boulevard, Baltimore, Maryland 21239, USA; 4 Division of Hematology/Oncology, American University of Beirut Medical Center, PO Box 11-0236, Riad El-Solh 1107-2020 Beirut, Lebanon

**Keywords:** Embolism, Endocarditis, Nonbacterial, Noninfective, Stomach neoplasms, Thrombosis

## Abstract

**Introduction:**

Nonbacterial thrombotic endocarditis is a very rare complication of malignancy and other hypercoagulable states. It describes the deposition of small sterile smooth or verrucoid vegetations on the valve leaflets, causing a clinical picture similar to that of bacterial endocarditis. The authors reported this case because this is a rare and unusual first manifestation of malignancy generally and of gastric cancer particularly, with only a few reports present in the literature.

**Case presentation:**

The authors present a case of a 36-year-old Caucasian male with occult gastric cancer whose first and only manifestation was nonbacterial thrombotic endocarditis causing extensive multi-organ infarctions.

**Conclusions:**

An endocarditis not responsive to antibiotics should raise the suspicion of an occult malignancy. Differentiating between an infective endocarditis and a nonbacterial thrombotic endocarditis will cause a radical change in the management which will eventually affect the patient’s prognosis.

## Introduction

Nonbacterial thrombotic endocarditis (NBTE) is a rare entity that describes small sterile vegetations on normal heart valves in the absence of a bacterial infection in the blood [1-3]. This condition most commonly affects the aortic valve and to a lesser extent the mitral valve or both and more rarely the right-sided valves [4].

In this case report we present a 36-year-old Caucasian male, previously asymptomatic, who visited our hospital with a picture of endocarditis, which turned out to be nonbacterial and the first manifestation of occult gastric cancer.

The association between cancer and thrombophilia is well known and described in the medical literature. As reviewed by el-Shami *et al.*, it is estimated that thromboembolic events occur in about 15% of patients with cancer, and evidence of venous thromboembolism will appear on postmortem examination in 50% of patients with an underlying malignancy [1]. Cancer-associated thrombophilia most commonly presents as venous thromboembolism, but it can also take the form of migratory superficial thrombophlebitis (described by Trousseau in 1865), arterial thrombosis, disseminated intravascular coagulation, and NBTE which was originally described by Ziegler in 1888 [1,2].

## Case presentation

A 36-year-old Caucasian male presented to our hospital after an episode of presyncope. He felt dizzy while in the bathroom and stepped out and lay down for 15 minutes before the dizziness subsided. He reported no previous similar episodes, as well as no prior history of any seizure-like activity, postictal state, or incontinence. He denied weight loss, night sweats, and high grade fever but reported one episode of chills a few hours prior to presentation. He directly presented to our Emergency Department (ED) to be worked up. One week prior to presentation he was admitted to an outside hospital for chest pain. He reported that the workup, including cardiac catheterization, done there was negative for any cardiac cause, but an ultrasound of his abdomen showed ascites. This latter finding prompted him to seek the advice of one of our gastroenterologists who saw him and ordered a computed tomography (CT) scan of his abdomen and pelvis.

**Figure 1:**
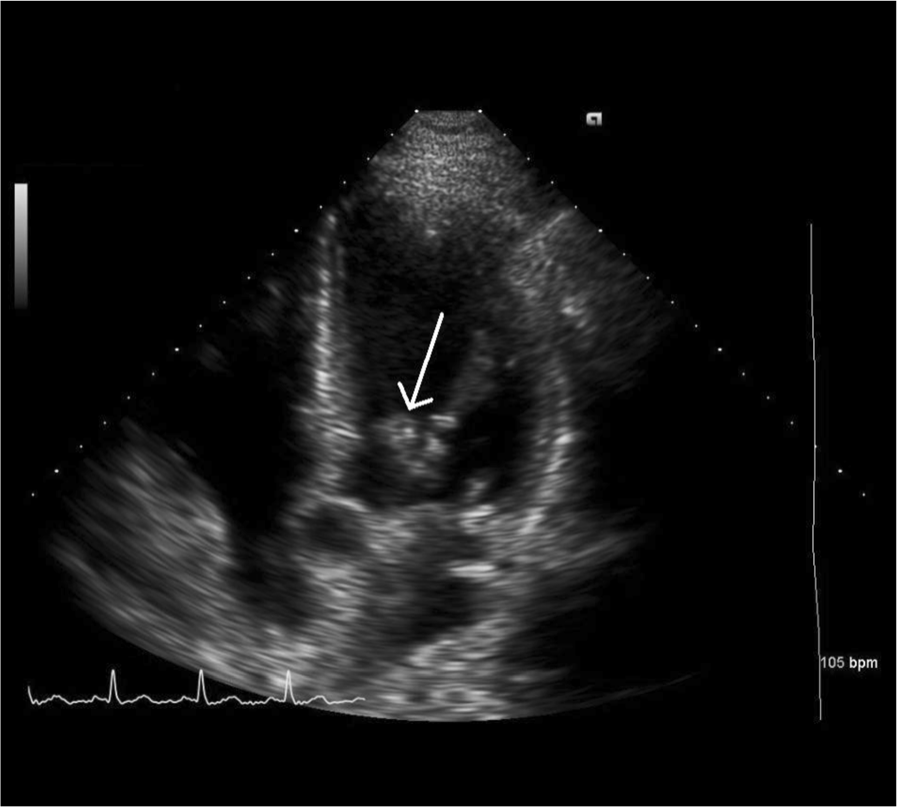
Transthoracic echocardiogram showing a mitral valve leaflet vegetation (arrow).

In the ED, his physical examination was significant for mild tachycardia, soft grade II/VI systolic ejection murmur on the left sternal border, and splinter hemorrhages in his nail beds. The rest of his physical examination, including neurologic examination, was normal. Portable transthoracic echocardiography (TTE) showed a mitral valve vegetation measuring 0.8cm×0.6cm (Figure 1). A CT scan of his abdomen and pelvis showed multiple lytic bone lesions, splenic and renal infarcts, evidence of malignant ascites, as well as omental caking. He was started on intravenous penicillin G 2 million units every 4 hours and gentamicin 80mg every 8 hours for presumptive bacterial endocarditis, while workup for hypercoagulable state and malignancy was taken. The hypercoagulability panel included D-dimer, international normalized ratio, lactate dehydrogenase (LDH), anticardiolipin immunoglobulin (Ig) G and IgM, antithrombin III, protein C, protein S, methylenetetrahydrofolate reductase (*MTHFR*) mutation screening, and lupus anticoagulants. The panel was significant for elevated D-dimer and LDH, heterozygosity for *MTHFR* mutation, and positive lupus anticoagulant, whereas the rest of the panel was within normal limits. In addition, antinuclear antibodies, and anti-B2 glycoprotein IgG and IgM were also negative. As for the malignancy workup, tumor markers were taken and were significant for elevated carcinoembryonic antigen and CA 19–9, whereas alpha-fetoprotein and prostate-specific antigen were both normal.

He was sent to undergo fine-needle aspirate (FNA) of the omental cake a few days later, and the FNA result showed adenocarcinoma. Colonoscopy was done and was normal, but esophagogastroduodenoscopy showed a gastric ulcer at the greater curvature in the gastric body from which biopsies were taken. The pathology results showed undifferentiated gastric adenocarcinoma.

A few days later he underwent a brain magnetic resonance imaging (MRI) based on the recommendations of the neurology team; they were worried that because he had a picture of multiple sites of infarction due to emboli from the site of endocarditis, he might also have showering of emboli to his brain, thus explaining his initial presentation with dizziness. His brain MRI showed an acute right middle cerebral artery stroke.

With the suspicion of bacterial endocarditis subsiding in light of his malignancy and hypercoagulability, and with his limited response to antibiotics, he was started on the low-molecular-weight heparin tinzaparin to treat his NBTE and overall hypercoagulable state, and he was followed up by the oncology team for chemotherapy. Serial blood cultures remained negative, and he was thus shifted from penicillin G and gentamicin to ciprofloxacin, gentamicin, and vancomycin treating possible culture-negative bacterial endocarditis. However, his serial blood cultures remained negative after 17 days of incubation, and all antibiotics were stopped and he remained on anticoagulation with the diagnosis of NBTE.

## Discussion

This is a case of NBTE secondary to metastatic gastric cancer (stage IV) in a previously healthy young man. He has previously had no symptoms like weight loss, fever, night sweats, or change in appetite or bowel movements. An infectious cause for his endocarditis was ruled out after consistently negative serial cultures, limited response to antibiotics and in view of his underlying malignancy. Even though the workup for a hypercoagulable state was positive for lupus anticoagulant and heterozygosity for the *MTHFR* mutation, these findings do not explain his presentation, especially since his family history is also negative for thrombotic events.

The pathogenesis of NBTE is not fully understood. One hypothesis incriminates interleukins and tumor necrosis factor (TNF), released secondary to the interaction between monocytes or macrophages and malignant cells [5,6]. This release of interleukins and TNF will cause endothelial damage and sloughing, which may lead to thrombogenesis on valvular surfaces as well as other sites in the bloodstream [5,6]. Platelets and clotting factors may also be activated by this interaction which could further facilitate thrombosis [5].

These sterile vegetations consisting of degenerating platelets and fibrin strands may have a wide range of sizes, and the absence of an inflammatory reaction at the site of attachment makes them very friable, with a tendency to detach more readily than those present in infective endocarditis [1-3,7]. This may lead to infarcts in various organs, including the spleen, kidney, heart and brain [6,8]. In fact, stroke is commonly the first clinical presentation of NBTE [7].

The true incidence of NBTE is not known [1]. It can occur in any type of cancer except brain tumors [9]. Cancers of the lung, pancreas, and stomach, in addition to adenocarcinomas of unknown primary sites, are the most common malignancies associated with NBTE [10]. Infective endocarditis and NBTE have many common risk factors. Hence it is often possible to clinically confuse these two entities [2].

According to a review by el-Shami *et al.*, no signs or symptoms are specific for NBTE, but a murmur in a patient known to have a malignancy should warrant the clinician to consider NBTE on the differential diagnosis. In general, if endocarditis is suspected in a patient known to have a history of malignancy and he/she has had no significant response to antibiotics, the physician should consider NBTE [1]. Similarly to infective endocarditis, transesophageal echocardiography has been shown to be more sensitive in detecting vegetations than TTE [1,11].

Studies have shown that diffusion-weighted imaging (DWI) MRI may help differentiate cardioembolic strokes secondary to infective endocarditis from those secondary to NBTE. In an imaging study done in 2002, four patterns on DWI have been described in patients experiencing recurrent embolic strokes due to bacterial endocarditis or NBTE [7]. These patterns are the following: pattern 1, a single lesion corresponding to a solitary embolus; pattern 2, multiple closely spaced lesions in a single arterial territory; pattern 3, multiple punctuate disseminated lesions; and pattern 4, multiple small and medium or large disseminated lesions [7]. Those secondary to NBTE exhibit pattern 4, described as multiple small and medium or large disseminated lesions, whereas strokes secondary to infective endocarditis manifest as a mix of all four patterns [7].

Once NBTE is diagnosed, the two primary goals should be the treatment of the underlying malignancy and systemic anticoagulation [1]. Among all forms of anticoagulation, unfractionated heparin (subcutaneous or intravenous) has been shown to be the most effective in reducing the incidence of embolic events. Low-molecular-weight heparin has also been used and appears to be effective. Vitamin K antagonists such as warfarin should not be used as they seem to be ineffective in controlling the coagulopathy in NBTE [1,5]. The reason for that remains unknown. Patients with NBTE should stay on anticoagulation indefinitely [1].

In patients with potentially curable cancer and tumor-associated coagulopathy, tumor resection as a first therapeutic priority to eliminate the cause of the coagulopathy should be considered [12]. In cases of persistent embolic events despite anticoagulation and a potentially curable cancer, valvular surgery can be considered prior to tumor resection to minimize the damage caused by the recurrent emboli [12].

## Conclusions

Endocarditis in a patient of any age group, especially one who is nonresponsive to antibiotics, should raise concern for other etiologies such as occult malignancy. It is possible that malignancy might first manifest with signs and symptoms of thromboembolism, or rarely in the form of endocarditis. Given that the management of NBTE and bacterial endocarditis are different, this necessitates having the correct diagnosis.

## Consent

Written informed consent was obtained from the patient's next-of-kin for publication of this case report and accompanying images. A copy of the written consent is available for review by the Editor-in-Chief of this journal.

## Competing interests

The authors declare that they have no competing interests.

## Authors’ contributions

WS collected the data required for the case presentation and was a major contributor in writing the manuscript. AR worked on the literature review and was a major contributor in writing the manuscript. ES worked on the literature review and was a major contributor in writing the manuscript. AT was involved in all aspects of this case report. All authors read and approved the final manuscript.
